# Adaptive bandwidth of immunity: a systems-level framework for understanding immune regulation across time

**DOI:** 10.3389/fimmu.2026.1848870

**Published:** 2026-09-03

**Authors:** Razvan Constantin Ionitescu

**Affiliations:** MedArt Cliniq, Ramnicu-Valcea, Romania

**Keywords:** adaptive bandwidth of immunity, adaptive regulation, immune adaptability, immune regulation, immune resilience, immune-mediated disease, longitudinal immune dynamics, multi-omics

## Abstract

Immune-mediated diseases display substantial variability in clinical expression, treatment durability, relapse timing, and long-term trajectories that are not always fully explained by genetic predisposition, molecular pathways, or pharmacological exposure alone. Increasing observations from systems immunology suggest that immune behavior emerges through interactions across molecular, environmental, and temporal dimensions. This article introduces *Adaptive Bandwidth of Immunity (ABI)* as a conceptual systems-level framework describing the functional range within which immune regulatory networks preserve adaptive responsiveness, proportionality, reversibility, and coordinated recovery across biological perturbations. ABI is not proposed as a discrete biological pathway or a directly measurable variable. Instead, it is introduced as an emergent property inferred through longitudinal clinical trajectories, temporal response dynamics, and integrated biological observations. The framework draws upon concepts from immune homeostasis, trained immunity, immune tolerance, systems immunology, and environmental modulation while emphasizing preservation of adaptive flexibility across time. Within this interpretation, immune-mediated disease may be viewed not only through dysregulated activation but also through progressive restriction of adaptive regulatory capacity. The framework generates empirically approachable predictions and outlines potential directions for future operationalization through longitudinal cohorts, trajectory-based analyses, and integration of multi-omics with temporal clinical data. ABI is presented as a hypothesis-generating framework intended to support future investigation into adaptive regulation and dynamic immune behavior.

## Highlights

- ABI is introduced as a systems-level framework describing adaptive immune responsiveness across time.
- ABI is conceptualized as an emergent property inferred through longitudinal trajectories rather than direct scalar measurement.
- Immune-mediated disease may additionally reflect progressive restriction of adaptive regulatory flexibility.
- The framework integrates concepts from immune homeostasis, trained immunity, environmental modulation, and systems immunology.
- ABI generates empirically approachable predictions and outlines pathways toward future operationalization.

## 1 Introduction

The immune system is remarkably stable, yet it is never static. Throughout life, every infection, environmental exposure, dietary shift, therapeutic intervention, and inflammatory challenge leaves a biological imprint. 

Nevertheless, immune regulation usually preserves coherence, proportionality, and the capacity to recover. Stability therefore emerges not from biological immobility, but from continuous adaptation. Clinical observation reminds us that this stability is never absolute. Patients with apparently similar diseases, comparable molecular profiles, and equivalent therapeutic interventions often experience profoundly different biological futures. 

Some remain in sustained remission for years. Others relapse unexpectedly. Some progressively lose immune control, whereas others recover despite persistent biological abnormalities. Modern immunology has transformed our understanding of the molecular mechanisms underlying these trajectories. 

Yet molecular precision alone does not fully explain how immune behavior unfolds across time.Perhaps the most remarkable property of the immune system is not that it adapts.It is that it continues to adapt. The central biological question is no longer simply what becomes dysregulated, but how much coordinated adaptive capacity remains available as immune regulation evolves across time. The ABI framework is proposed as one possible organizational perspective through which that question may be explored.

This perspective builds on an increasingly recognized view of the immune system as a dynamic, multi-layered regulatory network integrating molecular signalling, cellular interactions, and environmental inputs to preserve functional stability across changing physiological conditions (1–3). Rather than operating through isolated pathways, immune responses are increasingly interpreted as arising from coordinated interactions across multiple regulatory layers, a perspective central to systems immunology (2, 4, 5). Despite remarkable advances in our understanding of immune mechanisms, several clinically observed phenomena remain insufficiently explained within existing conceptual frameworks. These include delayed disease relapse following therapy withdrawal, differences in therapeutic durability among patients with similar diagnoses, and variability in the relationship between genetic susceptibility and phenotypic expression across immune-mediated diseases (6–8). Classical immunological paradigms—including immune homeostasis and tolerance—have provided important conceptual foundations by emphasizing balance, regulation, and controlled responsiveness (9, 10). However, these frameworks have historically emphasized relatively stable regulatory equilibria and may not fully capture the adaptive flexibility of immune behavior under continuous perturbation. Recent work has highlighted substantial inter-individual variability in immune responses, demonstrating that immune phenotypes may differ considerably even among genetically related populations (6, 11–14). These differences are shaped not only by intrinsic determinants but also by environmental exposures, microbial diversity, and context-dependent regulatory influences (15–19). At the molecular level, key signaling pathways—including NF-κB, JAK–STAT, and PI3K—govern immune activation, intercellular communication, and adaptive regulation (20–23). Instead of functioning independently, these pathways operate within interconnected regulatory networks whose emergent behavior is shaped by context-dependent interactions (2, 18). Emerging evidence from epigenetic conditioning and long-term immune programming further suggests that immune responsiveness reflects cumulative regulatory processes extending across previous exposures and dynamic recalibration over time (24–27). Population-level observations—including regional divergence in immune-mediated disease prevalence despite similar genetic backgrounds—further underscore the importance of environmental and adaptive regulatory influences in shaping immune trajectories (17). Taken together, these observations support exploration of conceptual models capable of integrating molecular mechanisms, environmental influences, and longitudinal clinical trajectories into a unified systems-level interpretation of immune function. Within this context, we propose the concept of the *Adaptive Bandwidth of Immunity (ABI)* as a systems-level framework describing the dynamic capacity of immune networks to preserve adaptive responsiveness across perturbations (2, 18). Beyond focusing on static equilibrium states or individual signaling pathways, ABI conceptualizes immune function as a flexible adaptive range characterized by regulatory flexibility, responsiveness, recovery dynamics, and context-dependent regulation. In this view, observed *clinical* variability may reflect differences in adaptive capacity across individuals and conditions rather than merely random biological variation. Immune behavior may emerge from coordinated interactions between molecular signaling, environmental modulation, and temporal system organization. ABI does not replace established immunological paradigms; instead, it provides an integrative structure through which homeostasis, tolerance, immune memory, and environmental conditioning may be interpreted within a common systems-level perspective (2, 4, 9, 24, 25). By integrating mechanistic observations with clinical trajectories, the ABI framework aims to offer a conceptual basis for understanding immune-mediated disease not as a static dysfunction, but as a dynamic process unfolding within constrained adaptability—a perspective explored throughout the following sections and revisited in the concluding synthesis (28–30).

## 2 Clinical observations supporting dynamic immune adaptability

Clinical practice provides a range of observations that are difficult to reconcile with linear or pathway-centered models of immune regulation. Across multiple immune-mediated diseases, patterns of disease activity, remission, and relapse exhibit temporal dynamics that are not fully explained by pharmacokinetic, genetic, or single-pathway explanations (7, 8, 31–33).

### 2.1 Temporal dissociation between drug exposure and disease reactivation

One of the most consistent findings in clinical practice is delayed disease reactivation following discontinuation of targeted therapies when patients remain in remission. In patients treated with Janus kinase (JAK) inhibitors for conditions such as rheumatoid arthritis, psoriatic arthritis, and spondylarthritis, disease relapse frequently occurs weeks to months after treatment withdrawal despite relatively short drug half-lives and minimal immunogenicity (7, 20, 33).

This temporal dissociation between pharmacokinetics and clinical relapse is consistent with the possibility of persistent regulatory processes operating beyond immediate pharmacological exposure. Patients who achieve sustained remission or low disease activity often may remain clinically stable after cessation of expected pharmacological exposure, indicating that immune regulation may persist independently of direct pharmacological inhibition. Such observations are difficult to reconcile with models based exclusively on pathway blockade and instead suggest the existence of system-level regulatory capacity contributing to response durability (28, 31).

### 2.2 Heterogeneity of clinical trajectories

A defining characteristic of immune-mediated diseases is substantial variability in clinical trajectories among patients with similar diagnoses. In clinical practice, some individuals maintain prolonged remission, whereas others experience early relapse or fluctuating disease activity despite comparable therapeutic interventions (8, 34, 35). Real-world registry data further highlight this heterogeneity. Observational cohorts in rheumatoid arthritis, psoriatic arthritis, and spondylarthritis demonstrate marked inter-individual differences in treatment response, durability, and relapse dynamics (36–38). Together, these findings suggest that clinical outcomes are determined not only by disease classification or therapeutic target selection, but also by differences in adaptive trajectories that shape how immune systems may additionally reflect differences in longitudinal immune regulation over time.

### 2.3 Non-linear transitions between stability and flare

Clinical evolution in immune-mediated diseases is frequently characterized by non-linear transitions. Patients may remain clinically stable for prolonged periods and subsequently experience abrupt disease flares or demonstrate rapid improvement following intervention without gradual progression (7, 27). Such patterns are consistent with *threshold-dependent dynamics* observed in complex biological systems, where relatively small perturbations may trigger disproportionate responses once regulatory limits are exceeded (1, 5). These observations suggest that immune regulation may operate within constrained adaptive states, where apparent clinical stability can persist despite accumulating perturbations until transition thresholds are reached. Such transitions may occur without proportional changes in conventional biomarkers, emphasizing the importance of longitudinal rather than static interpretation of immune behavior. This behavior suggests that disease activity is governed not only by inflammatory triggers but also by the adaptive capacity of the immune system to absorb, regulate, and recover from perturbations.

### 2.4 Convergence across diseases and therapeutic contexts

Similar clinical patterns are observed across different diseases and therapeutic classes.

Delayed relapse, heterogeneous treatment response, and non-linear disease transitions have been described across rheumatoid arthritis, psoriatic arthritis, spondylarthritis, and additional immune-mediated conditions (7, 31–33). This convergence suggests that these observations may not be restricted to individual pathways or disease categories but instead may reflect common regulatory features of immune regulation. Such convergence supports exploration of broader systems-level interpretations capable of integrating disease-specific mechanisms with adaptive immune behavior.

### 2.5 System-level interpretation

Taken together, these observations complement models in which immune behavior is determined exclusively by antigen recognition, genetic predisposition, or isolated signaling pathways. Instead, they support interpretation of immune behavior as an emergent property arising from interactions among regulatory networks over time (1, 2, 5).

The persistence of controlled responses after therapy withdrawal, variability in relapse timing, and non-linear transitions collectively suggest the existence of an adaptive regulatory range within which immune responses remain coordinated and reversible (2, 18).

Within this context, these clinical observations can be interpreted as system-level manifestations of differences in adaptive regulation. Rather than defining disease behavior through isolated molecular events, they support the view that immune outcomes emerge from interactions between regulatory flexibility, responsiveness, recovery potential, and environmental context (9, 26). This interpretation provides the conceptual foundation for the ABI framework developed in the following sections.

## 3 The adaptive bandwidth of immunity

### 3.1 Conceptual definition

**Figure 1:**
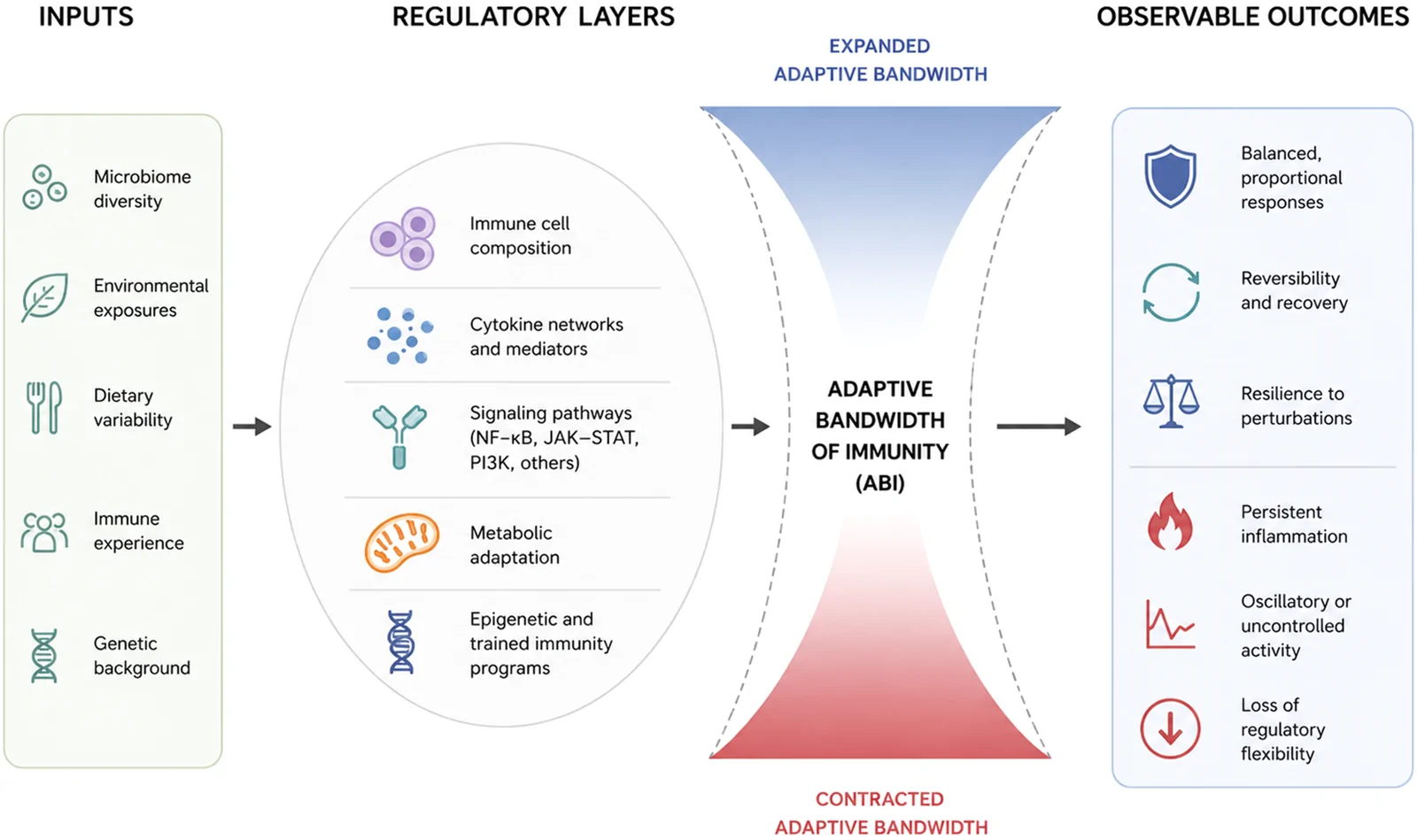
Conceptual representation of adaptive bandwidth of immunity (ABI).The image illustrates ABI as a systems-level property describing the functional range through which immune regulatory networks maintain balanced, proportional, and reversible responsiveness across changing internal and external conditions. Environmental and experiential inputs—including microbiome diversity, environmental exposures, dietary variability, cumulative immune experience, and genetic background—continuously shape immune calibration and adaptive potential. These influences are integrated through multiple regulatory layers comprising immune cell composition, cytokine communication networks, intracellular signaling pathways (including NF-κB, JAK–STAT, and PI3K-associated mechanisms), metabolic adaptation, and epigenetic regulatory programs. Collectively, these interconnected processes determine the adaptive bandwidth.

The ABI is conceptualized as a latent systems-level property describing the functional range within which immune regulatory networks maintain adaptive responsiveness, proportionality, and reversibility across internal and external perturbations (9, 10, 26). Instead of representing a directly measurable scalar variable, ABI reflects an emergent functional state that can be inferred through longitudinal clinical trajectories, temporal response patterns, and multi-scale biological correlates (18, 19, 39). In contrast to static constructs such as immune homeostasis, which primarily describe equilibrium states, ABI captures the dynamic range within which immune responses remain coordinated, regulated, and capable of recovery following perturbation (9, 26). This adaptive range is not fixed but continuously shaped through interactions between signaling activity, cellular composition, metabolic conditions, environmental exposures, and prior immune experience (15, 20–25). From this perspective, immune behavior is interpreted not as isolated activation or suppression events, but as the capacity of the system to maintain functional responsiveness while preserving internal regulatory balance (10, 26). The conceptual framework of ABI is illustrated in **Figure 1**.

Expanded bandwidth is associated with preserved immune flexibility, resilience to perturbation, proportional response intensity, and recovery following challenge. In contrast, contraction of adaptive bandwidth is associated with persistent inflammatory activity, impaired reversibility, reduced regulatory flexibility, and increased susceptibility to unstable disease trajectories. In this model, ABI is interpreted not as an independent biological pathway but as an integrative systems descriptor linking environmental complexity, regulatory adaptability, and dynamic clinical behavior over time. Variations in adaptive bandwidth are associated with distinct functional states ranging from preserved immune flexibility to narrowed adaptability and progressive immune dysregulation. Immune function is therefore interpreted as a continuum of regulated states defined by the capacity to absorb perturbation, modulate response intensity, and restore stability over time (18, 26).

### 3.2 Functional characteristics of ABI

A preserved adaptive bandwidth is characterized by the ability of immune systems to maintain coordinated regulation despite changing environmental and biological conditions. Core functional characteristics of preserved ABI include:

- proportional response to perturbation intensity;
- preservation of regulatory feedback integrity;
- reversibility following immune activation;
- resilience to repeated environmental and inflammatory stressors;
- maintenance of stable functional states across time (10, 22, 26).

These characteristics are interpreted as emergent system behaviors rather than discrete molecular measurements and therefore cannot be reduced to individual biomarkers or isolated signaling outputs (2, 18). When adaptive range becomes reduced, immune responses may become exaggerated, prolonged, insufficiently controlled, or less capable of returning toward stable regulation. Such contraction may manifest clinically as persistent inflammation, increased susceptibility to dysregulation, unstable disease trajectories, incomplete resolution, or reduced therapeutic durability (6–8, 31–33, 40). These transitions may be interpreted as progressive shifts across functional states, with progressive narrowing of adaptive capacity preceding overt instability. This perspective aligns with observations from systems immunology suggesting that coordinated immune behavior emerges from interactions among multiple regulatory layers rather than from isolated pathway activation (2, 6, 18). Accordingly, ABI is interpreted as reflecting not the magnitude of immune activation itself, but the conditions under which immune activation remains adaptable, reversible, and context dependent. To provide an integrative overview of determinants, regulatory mechanisms, and clinical consequences shaping ABI, these elements are summarized in **Table 1**.

**Table 1:** Determinants, regulatory dimensions, operational correlates, and clinical manifestations associated with adaptive bandwidth of immunity (ABI).

Determinant	Regulatory dimension	Potential operational correlate	Possible clinical manifestation
Environmental diversity	Expanded adaptive responsiveness	Stability across longitudinal measurements	Sustained remission
Microbiome exposure	Preservation of regulatory flexibility	Reduced fluctuation of immune trajectories	Lower relapse frequency
Prior immune experience	Adaptive recalibration	Delayed disease reactivation	Durable treatment response
Cytokine network organization	Preservation of signaling proportionality	Stable response kinetics	Controlled inflammatory activity
JAK–STAT signaling plasticity	Context-dependent immune modulation	Variable treatment durability	Differential therapeutic response
NF-κB regulatory balance	Controlled inflammatory amplification	Reduced inflammatory persistence	Lower risk of chronic activation
Epigenetic conditioning	Long-term immune calibration	Sustained adaptive responsiveness	Sustained recovery capacity
Regulatory feedback integrity	Maintenance of reversibility	Recovery following perturbation	Prevention of prolonged flare
Integrated regulatory coordination	Coordinated system behavior	Reduced trajectory fragmentation	Greater clinical stability
Adaptive regulatory reserve	Preservation of functional range	Recovery kinetics across time	Resilience to immune perturbation

ABI, Adaptive Bandwidth of Immunity; JAK–STAT, Janus kinase–signal transducer and activator of transcription; NF-κB, Nuclear factor kappa B.

Interpretation: Adaptive bandwidth is conceptualized as an emergent systems property inferred through interactions among environmental determinants, regulatory processes, and longitudinal immune behavior rather than through isolated molecular measurements.

### 3.3 Mechanistic basis of adaptive bandwidth

At the molecular level, ABI reflects the dynamic balance emerging from interactions among multiple signaling pathways and regulatory processes rather than the activity of any isolated biological mechanism. Contemporary immunology increasingly recognizes immune regulation as an emergent property of interconnected networks integrating activation, suppression, adaptation, and recovery across time (2, 6, 18). Viewed in this way, adaptive bandwidth is proposed to emerge from coordinated interactions among several regulatory domains.

*First*, inflammatory signaling pathways—including NF-κB-associated activation cascades—contribute to rapid immune responsiveness and amplification of host defense mechanisms (22).

Recent evidence further illustrates that dysregulated NF-κB signaling contributes to disease progression across diverse pathological settings beyond classical immune-mediated disorders. For example, WDR54-mediated enhancement of NF-κB activation has been shown to promote hepatocellular carcinoma progression, highlighting how perturbation of conserved regulatory signaling networks may influence complex biological system behavior across different diseases (41).

*Second*, adaptive signaling plasticity mediated through JAK–STAT pathways enable context-dependent regulation of cytokine responses and contributes to modulation of immune intensity and duration (20, 23).

*Third*, epigenetic and trained immunity mechanisms provide longer-term calibration of responsiveness by integrating previous immune exposures into future regulatory states (24, 25).

*Fourth*, environmental conditioning and host–microbiome interactions continuously reshape immune regulation and influence the range through which adaptive responses remain flexible (15–17).

These processes operate within interconnected regulatory networks whose system-wide regulatory flexibility contributes to *the system’s ability* to maintain coordinated responses without transitioning into persistent pathological states. Loss of this flexibility may contribute to persistence of activation, impaired resolution, oscillatory disease behavior, and progressive reduction in adaptive responsiveness (22, 26). Of note, ABI is not proposed as an independent biological pathway. Instead, ABI functions as an *integrative descriptor* of how multiple regulatory layers interact to preserve immune adaptability across changing biological contexts. This interpretation aligns with systems-level models in which disease behavior emerges from coordinated perturbation of network organization rather than isolated pathway dysfunction (1, 2, 5, 18). Disease-specific integrative models similarly illustrate how interacting vascular, inflammatory, and fibrotic processes may contribute to complex longitudinal disease behavior (42). In this context, immune dysfunction may be interpreted not simply as excessive activation or inadequate suppression, but as progressive narrowing of the functional range within which regulatory adaptation remains possible (18, 22, 26). This conceptual positioning distinguishes ABI from pathway-centered models while remaining fully compatible with contemporary mechanistic immunology.

#### 3.3.1 Mechanistic interpretation of ABI

Within this model:

- NF-κB contributes to inflammatory amplification (22)
- JAK–STAT contributes to adaptive signaling flexibility (20, 23)
- epigenetic conditioning contributes to longer-term calibration (24, 25)
- microbiome and environmental diversity contribute to regulatory breadth (15–17)
- systems integration determines observable immune trajectories (2, 5, 18)

These domains define the biological context through which adaptive bandwidth may emerge and become observable through longitudinal immune behavior.

### 3.4 Relationship to existing immunological models

The ABI framework builds upon established concepts in immunology while introducing a systems-level interpretive perspective. Rather than replacing existing paradigms, ABI seeks to organize and integrate complementary observations within a framework emphasizing dynamic adaptability across time (2, 18). Contemporary immunology increasingly recognizes immune function as emerging from interconnected molecular, cellular, and environmental interactions operating across multiple temporal scales (1–3, 6, 18). From this viewpoint, ABI contributes a specific focus on the functional range and adaptive responsiveness of immune regulation, linking molecular mechanisms with longitudinal clinical behavior.

#### 3.4.1 Relationship to immune homeostasis

Immune homeostasis traditionally describes maintenance of functional balance between activation and regulation and remains central to understanding immune stability (9, 26).

Although often represented conceptually as equilibrium, homeostasis also accommodates dynamic regulation across changing physiological conditions.

Within the ABI framework, stability is interpreted not as a fixed set point but as preservation of adaptive responsiveness across perturbations.

Thus, ABI extends rather than opposes homeostatic concepts by emphasizing the dynamic range through which balance is preserved.

#### 3.4.2 Relationship to immune tolerance

Immune tolerance refers to mechanisms limiting inappropriate responses toward self-antigens and harmless environmental stimuli (27). Within the ABI framework, tolerance represents one regulatory component contributing to preservation of adaptive responsiveness. Loss of tolerance may therefore reflect both local regulatory failure and broader alterations in systems-level immune adaptability. Accordingly, immune tolerance is interpreted not as an isolated suppressive mechanism but as one component of coordinated regulation across immune states.

#### 3.4.3 Relationship to trained immunity and immune memory

Trained immunity and adaptive immune memory describe the capacity of immune systems to modify future responses based on prior exposure through epigenetic, metabolic, and cellular reprogramming (24, 25). These mechanisms introduce temporal structure into immune behavior and influence subsequent responsiveness.

ABI incorporates these observations but differs in conceptual scope. Where trained immunity primarily addresses modification of future responsiveness, ABI focuses on the broader adaptive range within which responses remain regulated, reversible, and context dependent. Trained immunity may therefore contribute to shaping adaptive bandwidth without independently define its functional boundaries.

#### 3.4.4 Relationship to the danger model

The Danger Model proposes that immune activation is initiated primarily by signals associated with tissue stress and damage rather than recognition of foreignness alone (10, 22). This framework substantially expanded understanding of immune initiation mechanisms. ABI addresses a complementary dimension by focusing on how immune responses evolve over time after activation. Where the Danger Model emphasizes conditions initiating immune responses, ABI emphasizes variability in persistence, recovery, regulation, and reversibility. These frameworks operate at different explanatory levels and therefore may be viewed as complementary rather than competing.

#### 3.4.5 ABI as an integrative systems framework

A defining feature of ABI is its *integrative orientation*. Instead of describing isolated pathways, ABI proposes a conceptual structure for interpreting how multiple determinants collectively shape adaptive responsiveness.

Relevant contributors include:

- molecular signaling networks
- cellular and regulatory interactions
- environmental conditioning
- longitudinal clinical trajectories
- temporal response dynamics

By integrating these components, ABI offers a systems-level perspective linking immune organization with observable disease behavior. Notably, ABI should not be interpreted as replacing established immunological paradigms but as providing an additional organizational layer through which existing concepts may be connected and interpreted across biological scales. Overall, this framework provides a systems-level language for relating molecular regulation, environmental modulation, and clinical trajectories within immune-mediated disease (1–3, 5, 6, 18).

### 3.5 ABI as a determinant of disease states

Within the ABI framework, immune-mediated diseases may be interpreted as states associated with contraction or distortion of adaptive bandwidth rather than as isolated molecular abnormalities. When adaptive bandwidth is preserved, immune responses remain proportional, context dependent, and capable of efficient recovery following perturbation (9, 26). Under these conditions, inflammatory activation remains reversible, regulatory feedback loops remain functional, and immune responses retain sufficient flexibility to adapt without progressing toward persistent instability (22, 23). Conversely, when adaptive bandwidth becomes progressively reduced, immune responses may become less predictable, less reversible, and more vulnerable to dysregulation. Such narrowing may manifest clinically through exaggerated inflammatory activity, incomplete resolution, recurrent disease flares, oscillatory disease behavior, reduced therapeutic durability, or increased sensitivity to environmental perturbations (7, 8, 31–33, 40). Of note, this interpretation does not imply that disease emerges from a single measurable loss of adaptability. Rather, ABI proposes that disease behavior may reflect progressive alteration in the functional range through which immune systems maintain coordinated regulation. Viewed through this lens, distinct immune-mediated conditions may share common systems-level properties despite divergent initiating mechanisms and molecular signatures. This interpretation aligns with emerging systems biology approaches suggesting that disease states often reflect network reorganization rather than isolated pathway failure (1, 2, 5). Taken together, disease heterogeneity may be interpreted not merely as variation in pathogenic mechanisms but also as *variation in adaptive capacity* across individuals and environments. This perspective may help explain why patients with similar diagnoses and comparable molecular profiles frequently exhibit markedly different clinical trajectories and therapeutic responses (6, 11–14). However, ABI should not be interpreted as replacing established pathophysiological models. Rather, it offers an additional systems-level dimension through which disease expression, persistence, and recovery may be interpreted. In this model, disease progression may reflect reduced capacity to maintain coordinated immune regulation not only as accumulation of pathological activation but also as progressive restriction of the system’s ability to remain adaptable under changing biological conditions. This interpretation positions *adaptive capacity as an organizational property* emerging across molecular, environmental, and temporal dimensions of immune regulation.

### 3.6 Testability and predictive value

A defining characteristic of the Adaptive Bandwidth of Immunity (ABI) framework is its ability to generate empirically approachable and potentially falsifiable predictions. Although ABI is introduced as a conceptual systems construct, it is not intended to remain purely descriptive. Instead, ABI is proposed as a latent functional systems property whose state may be inferred indirectly through longitudinal clinical trajectories, temporal response dynamics, and integrated biological observations (2, 18, 19, 39). This perspective aligns with systems biology approaches in which higher-order properties are reconstructed from coordinated behavior rather than measured directly through isolated variables (1, 2, 4, 5).

#### 3.6.1 Testable predictions

ABI should be interpreted as a conceptual framework with operational implications accessible through indirect inference rather than direct scalar quantification. From this conceptual model, several testable predictions naturally emerge.

##### 3.6.1.1 Prediction 1 – temporal dissociation between pharmacokinetics and clinical relapse

Delayed disease relapse following withdrawal of targeted therapies may associate more closely with preservation of immune regulatory capacity than with pharmacokinetic persistence alone. Under this interpretation, durable remission reflects not only suppression of inflammatory signaling but preservation of adaptive responsiveness across time (7, 8, 20, 31–33, 40).

##### 3.6.1.2 Prediction 2 – environmental modulation of adaptive capacity

Populations exposed to greater environmental and microbial diversity may demonstrate greater preservation of adaptive responsiveness despite comparable genetic susceptibility. This prediction is consistent with observations linking environmental conditioning with immune heterogeneity and variability of disease expression (15–17).

##### 3.6.1.3 Prediction 3 – restoration of adaptive flexibility

Therapeutic interventions capable of preserving or restoring adaptive regulation may alter relapse kinetics and recovery trajectories independently of immediate pathway inhibition. This prediction suggests that treatment durability may depend not only on pathway suppression but also on preservation of regulatory adaptability (4, 7, 28, 40).

##### 3.6.1.4 Prediction 4 – trajectory-based interpretation may provide greater predictive value than static classification

Longitudinal trajectories integrating clinical, molecular, and environmental variables may outperform static biomarkers in predicting immune stability, treatment durability, and future relapse. This prediction follow from the concept that adaptive regulation emerges across time rather than from isolated measurements (18, 19, 39).

#### 3.6.2 Potential operational proxies of ABI

Although ABI is not represented by a single biomarker, its effects may become indirectly observable through longitudinal adaptive behavior.

Potential operational proxies may include:

- temporal stability of inflammatory markers across repeated measurements
- recovery kinetics following physiological or therapeutic perturbation
- relapse-free interval after treatment withdrawal
- variability in treatment durability across individuals
- longitudinal fluctuation patterns in immune phenotypes
- integration of multi-omics and environmental exposure signatures (4, 18, 19, 28, 39, 40).

Under this framework, ABI is not interpreted as a fixed biological quantity but as an emergent property reflected through adaptive system behavior across time. Future work may determine whether combinations of these signals can approximate adaptive bandwidth and support future attempts at quantitative approximation.

#### 3.6.3 Toward validation

These predictions may be explored through longitudinal cohort studies, registry-based analyses, treatment withdrawal studies, and integration of multi-omics with temporal clinical datasets (4, 19, 28, 39, 40). ABI is not presented as a finalized biological law but as a hypothesis-generating systems framework intended to stimulate empirical investigation. Its scientific value therefore depends not on *immediate measurability* but on whether future evidence demonstrates reproducible relationships between adaptive regulation, clinical trajectories, and immune outcomes. A possible future direction may involve developing adaptability indices derived from longitudinal clinical and biological trajectories.

From this viewpoint, the central question shifts from identifying a single dysfunctional pathway toward understanding whether adaptive regulatory flexibility remains available when immune systems are challenged.

## 4 Genetic susceptibility and environmental modulation of adaptive bandwidth

### 4.1 Genetic predisposition and its limits

Genetic susceptibility represents an important component in the development of immune-mediated diseases. Genome-wide association studies (GWAS) have identified multiple loci associated with conditions such as rheumatoid arthritis, psoriatic arthritis, and other systemic autoimmune disorders, supporting the contribution of inherited factors to immune response variability (39, 43, 44).

However, genetic predisposition alone does not fully determine disease expression, as individuals with similar genetic backgrounds may display markedly divergent clinical trajectories ranging from sustained stability to severe disease manifestations (6, 8, 11, 14).

This variability suggests that additional regulatory layers contribute to translating inherited susceptibility into observed phenotype.

Within this context, the ABI framework suggests that immune adaptability may act as an intermediate regulatory layer influencing how genetic predisposition becomes clinically expressed.

### 4.2 Environmental conditioning of immune function

Environmental exposure plays a major role in shaping immune responsiveness throughout life. Microbial diversity, infections, dietary patterns, lifestyle factors, and environmental exposures may contribute to the continuous calibration and reorganization of immune function (15–19).

Reduced environmental diversity, particularly in highly urbanized settings, has been associated with increased prevalence of autoimmune and allergic diseases. In contrast, environments characterized by broader microbial exposure appear to support immune tolerance and regulatory balance (15–19).

A simplified overview of environmental determinants influencing adaptive bandwidth and their associated outcomes is provided in **Supplementary Figure 1**.

These observations support the interpretation that immune adaptability is not a fixed intrinsic attribute but a dynamic systems property responsive to environmental context.

### 4.3 The Karelia paradox as a model of immune modulation

A frequently cited example illustrating interaction between genetic background and environmental exposure is the Karelia region, where genetically related populations in Finland and Russia display substantially different prevalence of immune-mediated diseases (15–19).

Despite shared ancestry, individuals living in more industrialized environments exhibit higher frequencies of autoimmune and allergic conditions compared with populations exposed to greater environmental microbial diversity (15, 19).

This discrepancy is not fully accounted for by genetic background alone and is consistent with an important contribution of environmental conditioning to immune system behavior.

Within the ABI framework, these observations may be interpreted as reflecting differences in adaptive responsiveness. Exposure to diverse environmental stimuli may preserve broader adaptive responsiveness, whereas reduced exposure may contribute to narrowing of adaptive capacity and increased susceptibility to dysregulation.

### 4.4 ABI as a modulator of genetic expression

The ABI framework does not imply modification of genetic architecture but proposes that immune adaptability may influence how inherited susceptibility becomes clinically expressed. Rather than acting as an independent determinant, ABI provides a conceptual framework through which genetic susceptibility may be differentially expressed depending on environmental and regulatory context through interactions with environmental factors and immune regulatory networks. This conceptualization helps reconcile the apparent discrepancy between strong genetic associations and heterogeneous clinical expression observed across immune-mediated diseases. It also aligns with accumulating evidence suggesting that immune phenotypes emerge from the interaction between inherited factors and dynamic regulatory processes operating across time (6, 11–14). Thus, ABI may be interpreted as a systems-level intermediary linking inherited predisposition with longitudinal immune behavior.

### 4.5 Systems-level integration

From a systems perspective, the interaction between genetics and environment can be understood as a dynamic regulatory interaction in which immune adaptability functions as an intermediary layer. Instead of considering genetic and environmental influences as independent contributors, the ABI framework integrates them into a unified interpretive continuum linking inherited susceptibility, environmental modulation, and systems-level adaptability. This approach supports a more nuanced understanding of immune-mediated disease behavior by emphasizing variability, context dependence, and temporal dynamics alongside established risk-based interpretations. Taken together, these observations support ABI as a conceptual systems-level framework intended to organize interactions between inherited susceptibility, environmental modulation, and longitudinal immune behavior (1–6, 11–14, 17, 40, 45).

## 5 Testability and systems-level operationalization of adaptive bandwidth

### 5.1 From conceptual framework to testable model

A prerequisite for any systems-level framework is the ability to generate empirically testable and potentially falsifiable predictions. As introduced in Section 3.6, the ABI framework proposes that adaptive regulatory capacity may be inferred indirectly through longitudinal clinical trajectories, temporal response dynamics, and integrated biological observations rather than measured as a single biological variable. Within this perspective, operationalization does not seek to identify a unique biomarker. Instead, it aims to reconstruct higher-order adaptive system behavior by integrating complementary biological and clinical observations across multiple temporal scales. This approach is consistent with systems biology, where complex functional properties emerge from coordinated network behavior rather than isolated molecular measurements (1–5, 40, 45). Accordingly, the following sections outline practical avenues through which the ABI framework may eventually become operationally testable.

### 5.2 Testable predictions of the ABI framework

The detailed predictions introduced in Section 3.6 define the principal empirical directions through which ABI may be evaluated. Together, they suggest that longitudinal trajectories, environmental modulation, adaptive recovery, and cross-disease convergence represent complementary avenues for testing the framework rather than independent hypotheses.

### 5.3 Multi-omics integration as an operational approach

Recent advances in multi-omics technologies offer opportunities to approximate higher-order immune states through integrated biological observations. Combining transcriptomic, epigenomic, proteomic, metabolomic, and microbiome-derived information may enable reconstruction of coordinated immune states. An analogous systems-level strategy has emerged in oncology, where multi-omics integration is increasingly used to reconstruct complex regulatory networks and identify higher-order biological determinants that cannot be resolved through isolated molecular measurements.

Comprehensive analyses across multiple tumor types illustrate how coordinated interpretation of genomic, transcriptomic, epigenomic, and proteomic information provides a more complete understanding of disease organization than any individual molecular layer alone (46). Collectively, these developments support the broader principle that complex biological behavior emerges from interactions among multiple regulatory layers rather than from single molecular pathways. Although ABI is proposed within the context of immune regulation, similar systems-level strategies are already widely applied in oncology, providing a practical conceptual analogy for future operationalization of adaptive immune regulation.

Rather than being represented by a single biomarker, ABI may be conceptualized as a multidimensional systems profile reflecting:

- network connectivity and signaling coherence
- temporal response variability
- stability of regulatory feedback loops
- recovery capacity after perturbation
- persistence of adaptive responsiveness

This model aligns with systems-level models in which disease emerges from network perturbation rather than isolated molecular defects (2–5, 40, 45).

### 5.4 Longitudinal dynamics and clinical trajectories

A defining component of ABI operationalization is the integration of repeated clinical observations across time.

Longitudinal assessment allows characterization not only of disease state but of disease behavior.

Under this interpretation:

- preserved adaptive bandwidth is expected to associate with stable trajectories and controlled recovery;
- narrowing adaptive bandwidth may associate with increased variability and incomplete resolution;
- severe contraction may associate with unstable recurrent inflammatory activity.

**Figure 2:**
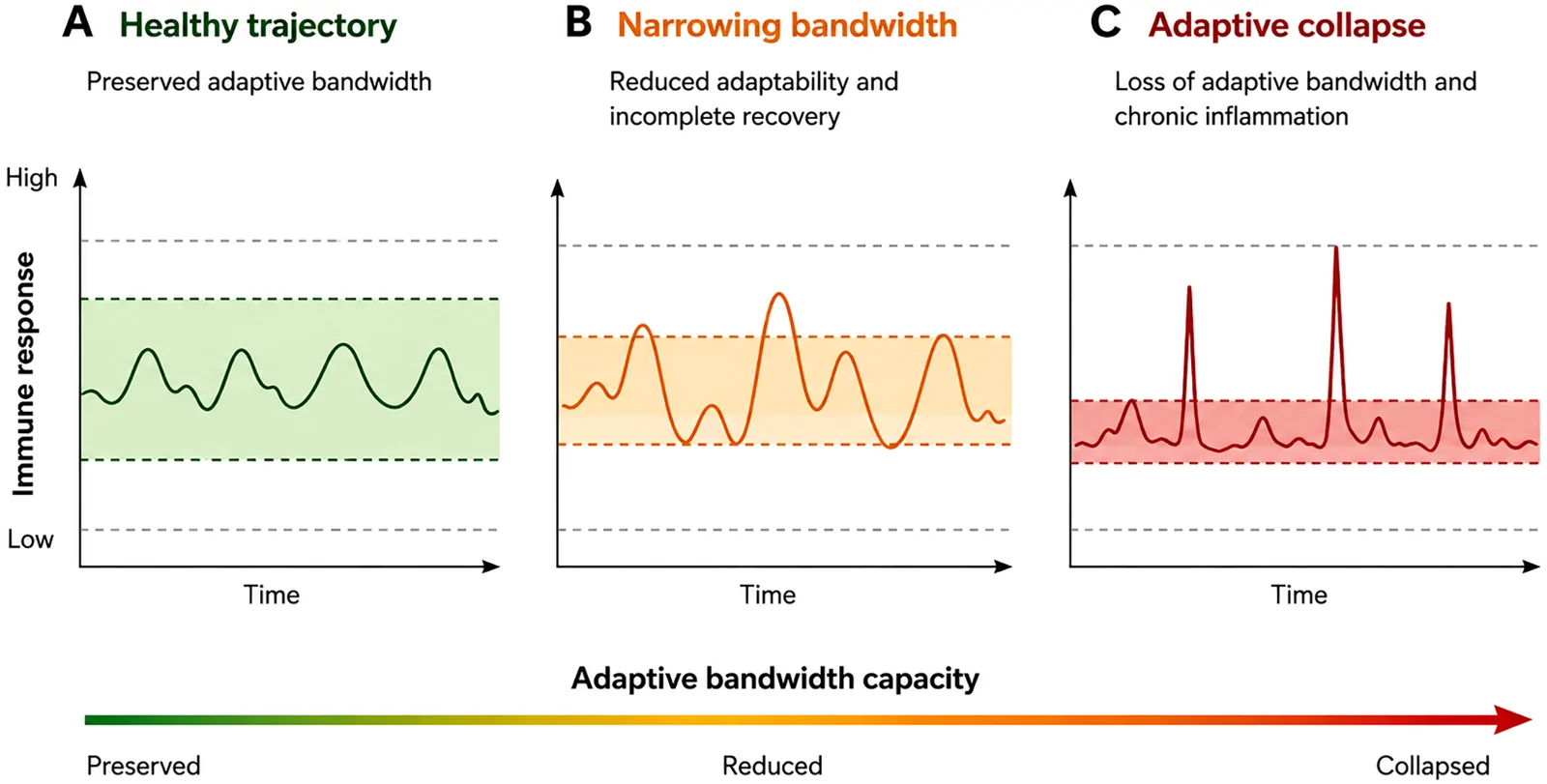
Immune response trajectories across adaptive bandwidth states. Representative temporal trajectories illustrating immune responsiveness across different states of Adaptive Bandwidth of Immunity (ABI). **(A)** Healthy trajectory (preserved adaptive bandwidth): immune responses fluctuate within a stable adaptive range, allowing proportional activation, efficient regulation, and recovery following perturbations. **(B)** Narrowing bandwidth (reduced adaptability): progressive restriction of adaptive capacity is associated with increased temporal variability, reduced regulatory buffering, incomplete recovery, and growing system instability. **(C)** Adaptive collapse: severe loss of adaptive bandwidth results in fragmented responsiveness, exaggerated fluctuations, and persistence of inflammatory activity with impaired return toward baseline. The horizontal gradient bar illustrates the conceptual transition from preserved to collapsed adaptive bandwidth capacity. The image is intended as a systems-level conceptual model and does not represent measured biological data or quantitative thresholds.

These trajectories should not be interpreted as discrete disease categories but rather as dynamic transitions across functional immune states (**Figure 2**). This view shifts analysis from static classification toward trajectory-based characterization.

#### 5.4.1 Conceptual interpretation

As illustrated in **Figure 2**, ABI is introduced as a conceptual systems framework describing the dynamic range through which immune networks maintain proportional responsiveness while preserving regulatory control. The trajectories shown are heuristic representations intended to illustrate differences in temporal organization of immune behavior rather than absolute biological states.

Narrowing of adaptive bandwidth should not be interpreted as a single molecular event but as an emergent property arising from interactions among immune regulation, environmental influences, and disease-specific mechanisms. Viewed from this perspective, disease expression reflects progressive constraints in adaptive capacity rather than isolated pathway activation (7, 8, 28, 29, 31–34, 36–38, 47–50).

### 5.5 Bridging clinical and systems-level data

The integration of longitudinal clinical observations with high-dimensional biological data provides a pathway toward practical implementation. Instead of relying exclusively on categorical disease labels, this framework emphasizes dynamic surrogate indicators of immune adaptability.

Potential readouts include:

- relapse timing
- variability of disease activity
- durability of treatment response
- recovery kinetics
- stability following perturbation

Such integration may support patient stratification based on functional immune behavior rather than phenotype alone. Taken together, these observations support ABI as a systems-level interpretive framework connecting molecular complexity with observable clinical trajectories (4, 28, 29, 36–38, 40, 45, 47).

## 6 Implications, limitations, and future directions

### 6.1 Toward an integrative interpretation of immune variability

ABI framework proposes a systems-level interpretation through which observations of immune variability may be organized across molecular, environmental, and clinical dimensions. Rather than assuming that immune-mediated disease reflects isolated pathway dysfunction, ABI considers the possibility that differences in disease expression may additionally reflect variation in adaptive regulatory capacity across time (1–8, 11–14, 31–33). This perspective may help contextualize observations frequently encountered in clinical practice, including prolonged remission following treatment withdrawal, heterogeneous therapeutic durability, divergent recovery kinetics, and variable disease trajectories among individuals with apparently comparable molecular profiles (4, 7, 8, 28, 31–33, 40). Within this interpretation, adaptive responsiveness is viewed not as a static property but as a dynamic regulatory range shaped through interactions among signaling networks, environmental exposures, prior immune experience, and longitudinal biological adaptation (15–27). Disease progression is therefore interpreted not simply as the accumulation of pathological activation, but as a potential reduction in the system’s capacity to preserve coordinated regulation under changing biological conditions.

### 6.2 Integration of genetic and environmental determinants

Current models increasingly recognize that immune-mediated disease emerges through interaction between inherited susceptibility and environmental modulation rather than through either domain alone (15–19, 39, 43, 44). Within this context, ABI introduces immune adaptability as a systems-level mediator linking biological predisposition with observable disease behavior. Under this formulation, genetic architecture is not replaced nor minimized. Instead, inherited susceptibility is interpreted within a broader regulatory environment shaped by microbial exposure, environmental complexity, cumulative immune experience, and dynamic network behavior (15–19, 39, 43, 44). This conceptualization may contribute to explaining why individuals with comparable genetic backgrounds frequently display divergent disease trajectories and variable treatment responses. Such variability may therefore reflect differences not only in pathogenic pathways but also in the preservation of adaptive regulatory flexibility.

### 6.3 Translational implications

From a translational perspective, the ABI framework encourages interpretation of therapeutic response beyond immediate pathway inhibition. Clinical improvement may reflect not only reduction of inflammatory intensity but also preservation or restoration of adaptive regulation across time (4, 7, 8, 28, 31–33, 40, 47). Under this interpretation, patient-specific temporal trajectories may become increasingly relevant alongside molecular classification and baseline disease activity.

Potential applications for future investigation include:

- longitudinal assessment of treatment durability
- trajectory-informed disease stratification
- integration of multi-omics with temporal clinical behavior
- identification of surrogate indicators of adaptive regulation
- development of trajectory-derived adaptability indices

These directions are presented as exploratory applications rather than immediate clinical implementation.

### 6.4 Conceptual boundaries and limitations

Several limitations should be acknowledged.

First, ABI is introduced as a conceptual and organizational framework rather than a validated biological construct.

Second, ABI is not proposed as a direct biomarker or a single measurable biological variable. Instead, it represents a systems-level organizational framework whose future validity will depend on reproducible relationships among coordinated biological and clinical observations.

Third, the present framework does not provide a predictive mathematical model and should not be interpreted as replacing established mechanistic immunology.

Finally, adaptive behavior may differ substantially across tissues, diseases, and biological contexts, limiting direct generalization. These limitations do not diminish the value of the framework but define conditions under which future validation may become meaningful (4, 18, 19, 39, 40, 45).

### 6.5 Future directions

Future studies should evaluate whether adaptive regulation can be operationalized through reproducible longitudinal approaches.

Priority directions include:

- prospective longitudinal cohorts
- integration of multi-omics and environmental exposures
- computational reconstruction of immune trajectories
- validation of trajectory-derived biomarkers
- derivation of trajectory-based adaptability indices

Such approaches may help determine whether adaptive regulatory capacity can be indirectly approximated through coordinated biological and clinical observations (4, 19, 39, 40, 45).

#### 6.5.1 Toward operational validation

A central challenge for future work will be determining whether systems-level adaptive behavior can be inferred reproducibly across independent datasets.

Possible approaches include longitudinal registries, treatment withdrawal studies, temporal immune profiling, and integration of biological observations across scales (4, 18, 19, 39, 40, 45).

From this perspective, validation should not be interpreted as confirmation of a predefined model but as evaluation of whether reproducible relationships emerge between adaptive regulation and observable disease behavior.

Accordingly, the scientific value of ABI depends less on conceptual novelty and more on its ability to generate empirically useful predictions.

### 6.6 Concluding perspective

Emerging observations across systems immunology and longitudinal immune research are increasingly compatible with the possibility that immune regulation may involve dimensions extending beyond static pathway-centered interpretations (1–6, 11–14, 18).

The Adaptive Bandwidth of Immunity framework is proposed as one possible systems-level language through which these observations may be organized and explored.

Within this view, immune health may not simply reflect absence of activation but preservation of adaptive regulation across changing biological conditions.

Similarly, immune-mediated disease may also be interpreted not only through accumulated dysfunction but through variation in the capacity to maintain coordinated responsiveness over time.

Ultimately, the scientific value of this framework will depend not on conceptual originality alone but on whether adaptive regulation can be operationalized, empirically evaluated, and linked to reproducible clinical trajectories. If immune regulation is dynamic rather than static, then understanding disease may depend not only on identifying what is disrupted, but also on understanding what adaptive capacity remains.

## 7 Adaptive capacity across time

### 7.1 Every immune system carries an adaptive history

The immune system is often described through its current biological state.

Clinical activity, circulating biomarkers, cellular phenotypes, transcriptomic profiles, and imaging findings all provide valuable snapshots of immune regulation at a particular moment in time. These measurements have profoundly improved our understanding of immune-mediated diseases and remain fundamental for both clinical practice and translational research (1–4, 28, 40). Yet no immune system begins at the moment it is observed.

Every immune response emerges from *a biological history* that has been continuously shaped by previous infections, environmental exposures, nutritional influences, therapeutic interventions, metabolic adaptations, aging, and countless inflammatory events (6, 11–19, 25–27). Each experience leaves a trace—sometimes transient, sometimes persistent—that contributes to the organization within which future immune responses will develop. From this perspective, immune regulation may be viewed not simply as the expression of present molecular activity, but as the cumulative consequence of lifelong adaptive experience.

ABI builds upon this observation. Rather than interpreting immune behavior as a sequence of isolated biological events, the framework considers every observed immune state as the temporary expression of an adaptive trajectory that has evolved across time. *The present* therefore cannot be completely separated from the biological path that generated it. This distinction carries important conceptual implications. Two individuals may present with remarkably similar clinical manifestations, comparable inflammatory biomarkers, or overlapping molecular signatures while arriving at these observations through profoundly different adaptive histories. Their immune systems may therefore possess different capacities for future adaptation despite appearing biologically similar at the moment of evaluation (6, 8, 11–14, 34, 35). Within the ABI framework, adaptive history is not viewed simply as accumulated clinical experience. It becomes part of the biological architecture of immune regulation itself.

### 7.2 Adaptive capacity is a moving biological property

Adaptive capacity cannot remain unchanged if the system that generates it changes continuously throughout life. The immune system is never exposed twice to exactly the same biological conditions. Every infection, every successful recovery, every therapeutic intervention, every metabolic adaptation, every environmental exposure, and every episode of sustained inflammation subtly modifies the context within which future immune responses will occur (6, 9–14, 17–19, 25–27). Most of these changes are individually too subtle to be recognized. Their cumulative effect, however, progressively reshapes the adaptive possibilities available to the system. Adaptive bandwidth should therefore not be viewed as a fixed biological attribute. It represents a dynamic property that continuously evolves throughout life, reflecting the interaction between inherited regulatory mechanisms and accumulated biological experience (1–4, 40). The capacity to adapt today is unlikely to be identical to the capacity that existed years earlier, just as it will not remain identical in the years to come. This evolution is unlikely to follow abrupt transitions. Instead, adaptive capacity probably changes gradually, through the continuous integration of countless biological events whose individual impact may appear modest but whose collective influence becomes increasingly significant over time (4, 8, 28, 34, 35, 40, 45).

This perspective also changes how biological stability is interpreted. Stability no longer represents the preservation of an unchanged immune state. Rather, it reflects the ability of the system to preserve coordinated adaptation while continuously reorganizing itself in response to changing biological conditions. Within the ABI framework, adaptive bandwidth is therefore understood not as *a destination* that is eventually reached, but as a dynamic biological property that remains in continuous evolution throughout life.

### 7.3 Adaptive trajectories: beyond biological snapshots

Clinical medicine frequently evaluates immune-mediated diseases through observations made at discrete moments in time. Disease activity scores, laboratory parameters, imaging findings, transcriptomic profiles, and even tissue pathology provide invaluable information about the current biological state of the patient (4, 28, 29, 36–38, 40, 48–50). Yet every measurement represents only a single point along a much longer adaptive journey. Phenotypic assessments in systemic sclerosis similarly illustrate how discrete clinical or biological measurements may characterize the patient at a given moment without capturing the full longitudinal evolution of disease (51). ABI proposes that immune regulation should be understood not only through biological snapshots but also through the trajectories that connect them. This distinction may fundamentally influence the interpretation of immune behavior. Two patients may appear remarkably similar during a single clinical assessment while following completely different adaptive trajectories. One trajectory may reflect progressive restoration of adaptive organization despite persistent inflammatory activity. Another may indicate gradual contraction of adaptive capacity despite temporary clinical improvement (4, 8, 28, 29, 34–38, 40, 45, 48–50). From this perspective, identical biological states do not necessarily imply identical biological futures. *The present* describes where the immune system is. The trajectory suggests where it is going. This distinction becomes particularly relevant in chronic immune-mediated diseases, where years of continuous adaptation frequently precede clinically detectable transitions (4, 8, 28, 29, 34–38, 40, 45, 47–50). Changes in adaptive bandwidth may therefore begin long before conventional clinical deterioration becomes apparent, while successful therapeutic intervention may restore adaptive organization before complete clinical remission is achieved. ABI invites a longitudinal interpretation of immune regulation in which direction becomes as informative as position. The biological meaning of an immune state depends not only on its current characteristics but also on the adaptive trajectory from which it emerged and the trajectory toward which it is evolving.

### 7.4 Adaptive reserve: the space between stability and failure

Adaptive systems rarely fail at the moment they encounter biological stress. Their resilience lies in the ability to absorb perturbation, reorganize internally, and preserve coherent regulation despite continuously changing conditions. This capacity may be viewed as an adaptive reserve (1–3, 5, 30). Adaptive reserve is not an additional biological mechanism, nor does it correspond to a single measurable molecular entity. It represents the functional space within which immune regulation can reorganize itself while maintaining adaptive coherence. Like adaptive bandwidth itself, this reserve is unlikely to remain constant throughout life. Successful immune regulation, effective therapy, and restoration of tissue homeostasis may preserve—or occasionally enlarge—the adaptive space available to the system. Persistent inflammation, repeated immune activation, chronic metabolic stress, aging, and cumulative tissue injury gradually consume this adaptive reserve, leaving the immune system with progressively fewer possibilities for future adaptation (9, 10, 20–27, 29, 36–38, 48–50). A reduction in adaptive reserve does not necessarily produce immediate clinical deterioration. An individual may remain clinically stable while the biological flexibility required to respond to future perturbations is gradually diminishing. Restoration of adaptive reserve may begin long before conventional clinical markers indicate substantial improvement (4, 8, 28, 29, 34–38, 40, 45, 47–50). This perspective offers one possible explanation for a familiar clinical observation. Patients with apparently similar molecular profiles and comparable disease activity often demonstrate strikingly different resilience when confronted with similar biological challenges (4, 8, 28, 29, 34–38, 40, 45, 47–50). Their observable immune characteristics may overlap, while the adaptive reserve supporting those characteristics differs considerably. Within the ABI framework, adaptive reserve emerges from the cumulative organization of immune regulation throughout life. It reflects how much adaptive flexibility remains available before the system begins to lose its capacity for coordinated biological adaptation.

### 7.5 Adaptive transitions: when biological organization changes direction

The evolution of immune regulation is unlikely to follow a continuous linear course.

Long periods of apparent stability are frequently interrupted by transitions during which the adaptive organization of the system changes direction. Some transitions are clinically evident. The onset of autoimmune disease, sustained remission, therapeutic failure, or recovery after effective treatment all represent recognizable turning points in the biological history of the immune system (4, 8, 28, 29, 34–38, 40, 45, 47–50). Many others are likely to remain clinically silent. Adaptive organization may continue to evolve for months or years before conventional clinical manifestations become apparent. During these periods, immune regulation may appear relatively stable while its capacity for future adaptation is gradually being reshaped beneath the surface of observable disease activity (4, 28, 29, 36–38, 40, 48–50). This possibility suggests that clinical change may represent the visible consequence of biological transitions that began long before they became clinically detectable. Likewise, successful therapy may initiate adaptive reorganization before measurable improvements are reflected in conventional disease activity scores. Restoration of adaptive capacity may therefore precede restoration of clinical phenotype. From the perspective of ABI, these transitions become as biologically meaningful as the stable periods that separate them. The history of an immune system is not defined only by the states it occupies, but also by the transitions through which it moves. Viewing immune regulation through adaptive transitions therefore shifts attention from isolated clinical events toward the continuous evolution of biological organization across time.

### 7.6 Why ABI matters

Scientific progress is often marked by the discovery of new mechanisms. Equally important, however, are the moments when existing knowledge is viewed through a different conceptual lens. Such changes rarely replace previous discoveries; instead, they reorganize them into a broader and more coherent biological narrative (1–5, 40). ABI is proposed in this spirit. It does not seek to redefine immune regulation, nor does it compete with established concepts that have profoundly advanced contemporary immunology. Instead, it asks whether these mechanisms may also be interpreted as components of a continuously evolving adaptive organization whose behavior extends beyond any single biological pathway. This perspective invites a subtle but important change in the way immune-mediated diseases are interpreted. Instead of viewing disease exclusively as the consequence of dysregulated mechanisms, ABI encourages us to ask how much adaptive organization remains available within the system, how that organization has been shaped by previous biological experience, and how it may continue to evolve in the future.

The emphasis shifts from isolated biological events toward the continuity that connects them. Disease activity, remission, relapse, therapeutic response, and recovery cease to represent independent clinical episodes. They become successive expressions of the same adaptive journey (4, 8, 28, 34, 35, 40, 45). Perhaps the greatest contribution of ABI is not the introduction of another immunological concept. It is *the invitation* to observe immune regulation as a living process whose organization is continuously rewritten throughout life. The immune system is remarkable not simply because *it adapts*. It is remarkable because *it continues to adapt*. Understanding how long that capacity can be preserved may ultimately become as important as understanding the mechanisms through which it operates. Perhaps, in the end, immunity is defined not only by what it can do, but by what it can still become.

## Data Availability

The original contributions presented in the study are included in the article/**Supplementary Material**. Further inquiries can be directed to the corresponding author.

## Glossary

Adaptive Bandwidth of Immunity ABI: a systems-level functional property describing the dynamic capacity of immune networks to maintain adaptive, proportional, and reversible responses under internal and external perturbations.
Immune Adaptability IA: the intrinsic capacity of the immune-metabolic system to preserve functional stability and coordinated responses under varying physiological and inflammatory conditions.
Nuclear factor kappa B NF-κB: a key transcription factor regulating inflammatory responses, cytokine production, and immune activation.
Janus kinase: signal transducer and activator of transcription JAK–STAT — a signaling pathway mediating cytokine-driven communication and gene expression in immune cells.
Phosphoinositide 3-kinase PI3K: a signaling pathway involved in cellular metabolism, survival, proliferation, and immune regulation.
Genome-wide association study GWAS: an analytical approach used to identify genetic variants associated with diseases across the genome.
WD repeat domain 54 WDR54: a WD repeat-containing protein implicated in the modulation of NF-κB signaling in the context discussed in the manuscript.
